# Strength and Water Interactions of Cellulose I Filaments Wet-Spun from Cellulose Nanofibril Hydrogels

**DOI:** 10.1038/srep30695

**Published:** 2016-07-28

**Authors:** Meri J. Lundahl, A. Gisela Cunha, Ester Rojo, Anastassios C. Papageorgiou, Lauri Rautkari, Julio C. Arboleda, Orlando J. Rojas

**Affiliations:** 1 Aalto University, Department of Forest Products Technology. P.O. Box 16300, 00076 Aalto, Finland; 2 Turku Centre for Biotechnology, University of Turku and Åbo Akademi University, 20520 Turku, Finland

## Abstract

Hydrogels comprising cellulose nanofibrils (CNF) were used in the synthesis of continuous filaments via wet-spinning. Hydrogel viscosity and spinnability, as well as orientation and strength of the spun filaments, were found to be strongly affected by the osmotic pressure as determined by CNF surface charge and solid fraction in the spinning dope. The tensile strength, Young’s modulus and degree of orientation (wide-angle X-ray scattering, WAXS) of filaments produced without drawing were 297 MPa, 21 GPa and 83%, respectively, which are remarkable values. A thorough investigation of the interactions with water using dynamic vapour sorption (DVS) experiments revealed the role of sorption sites in the stability of the filaments in wet conditions. DVS analysis during cycles of relative humidity (RH) between 0 and 95% revealed major differences in water uptake by the filaments spun from hydrogels of different charge density (CNF and TEMPO-oxidised CNF). It is concluded that the mechanical performance of filaments in the presence of water deteriorates drastically by the same factors that facilitate fibril alignment and, consequently, enhance dry strength. For the most oriented filaments, the maximum water vapour sorption at 95% RH was 39% based on dry weight.

Novel, high-performance materials from renewable resources have been the subject of intensive research during the last couple of decades. Particular attention has been directed to cellulose nanofibrils (CNF), owing to their renewable origin as well as excellent mechanical strength. Indeed, the Young’s modulus of a single cellulose nanocrystal (CNC) has been estimated to be in the range between 100 and 160 GPa^1^. However, when CNCs are assembled in a multiscale material, as in CNF, CNF bundles or larger CNF-based structures, such high values can only be approached by careful control of orientation or alignment^2^. Examples on the methods reported thus far to assemble CNF in oriented films^3^,^4^,^5^ and cellulose I filaments^6^,^7^,^8^,^9^,^10^,^11^ are presented in Fig. 1 with an Ashby plot of the specific or density-normalised strength and Young’s modulus.

As shown in Fig. 1, even the most successful attempts to orient CNF in films or nanopapers fail to reach the level of mechanical performance expected for the single building block of cellulose I (CNC), not even that of a natural fibre comprising highly aligned cellulose fibrils. Likewise, the same spinning techniques that are known to be effective in the alignment of macromolecules in solution or in a melt, work poorly in the case of filaments derived from colloidal dispersions of CNF (Fig. 1, inset). In this case, a major role is played by the relatively smaller slenderness or aspect ratio (*i.e.*, characteristic length/diameter) of CNF (25–500)^12^ compared to that of a polymer (*e.g.*, tens of thousands for a typical spinnable polymer chain). Additional effects that arise in the different spinning processes are listed in Table 1. Henceforth, we use the generic term “wet-spinning” despite the fact that our work concerns only a specific type that uses a colloidal dispersion or a hydrogel of CNF as a dope. Essentially, polymer spinning techniques rely on the drawability of melted or dissolved polymers. If spinning is applied to CNF colloidal dispersions, fibril alignment needs to be prompted by mechanisms that overcome the poor drawability arising from the inherently smaller CNF aspect ratio.

Successful CNF-based filaments could be used as reinforcement in composite materials or to develop yarns for textile structures. However, these applications demand resistance to water, which remains a challenge for unmodified cellulose fibrils^12^,^13^. Stabilisation of a CNF filament against water has been demonstrated by surface modification^11^ and cross-linking^9^. Still, water-stability and the interactions with water remain subjects that require critical attention, especially in the case of all-cellulose systems. The study presented here addresses, therefore, the influence of CNF hydrogel quality on the properties of the respective spun filaments. Firstly, we determine the influence of CNF hydrogel solid fraction and surface charge on the ability of the fibrils to orient and form strong filaments during wet-spinning. Secondly, we identify the effect of CNF surface charge on the stability of the spun filaments in the presence of water.

## Results

### Rheology of the Precursor Hydrogels

Shear flow is the most significant flow type during spinning^14^. The shear rheology of a hydrogel dope is therefore expected to determine, to a large extent, its spinnability as well as the orientation of the fibrils in the nozzle. As such, the apparent and complex shear viscosities were measured for both unmodified and TEMPO-oxidised CNF (TOCNF). In the case of CNF, the solids content of the hydrogels was varied between 1% and 10%. For TOCNF, only the solids content of 2% was used because this value was discovered to produce the best-performing filaments from CNF, as will be shown below. All hydrogel samples are henceforth denoted according to the type of cellulose nanofibrils used (CNF or TOCNF) with a given solids content (*e.g.*, ‘CNF 2% hydrogel’ refers to a hydrogel dope containing 2 w-% of CNF).

**Figure 2:**
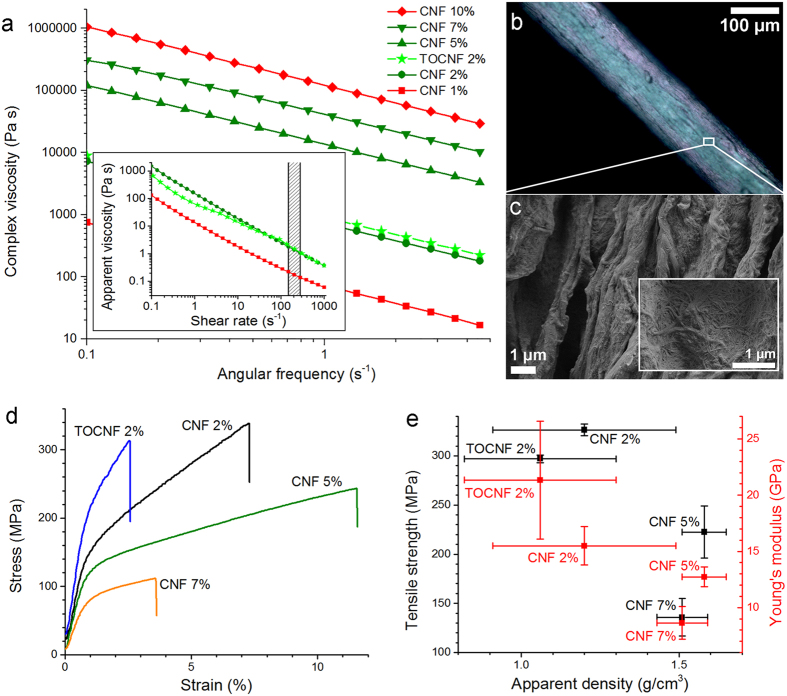
Rheological behaviour of hydrogel dopes and mechanical properties of the spun filaments. (**a**) Complex viscosity as a function of angular frequency for CNF and TOCNF hydrogels of given solids content. The inset displays the apparent viscosity as a function of shear rate for CNF 1–2% and TOCNF 2% hydrogels. The patterned zone indicates the approximate shear rate used in the spinning system. The spinnability of the hydrogels is indicated using a colour code: green for conditions spinning continuous filaments (length > 5 cm) and red for less spinnable, spun filaments < 5 cm. (**b**) Optical micrograph of CNF 2% filament between crossed polarisers. (**c**) SEM images of CNF 2% filament surface. (**d**) Representative stress-strain profiles for wet-spun filaments tested at RH 50%. In this condition, the equilibrium moisture of the TOCNF 2% filament is higher than those of the CNF filaments, as indicated by dynamic vapour sorption studies (DVS, see Fig. 5a-b). (**e**) Filament tensile strength and Young’s modulus as a function of the apparent density. Error bars correspond to the standard deviation among the tested specimens.

The apparent and complex shear viscosity of the hydrogels are shown in Fig. 2a. For systems at high CNF concentration (5–10%), only the complex viscosity is included since gel fracture prevents accurate determination of the apparent viscosity. For the systems at low solids content (1–2%), a shear-thinning behaviour was observed between 0.1 and 1000 s^−1^ (Fig. 2a, inset). Moreover, a higher apparent viscosity was measured as the CNF solid fraction in the dope was increased. The hydrogel complex viscosity (Fig. 2a) is approximately ten-fold higher than the apparent viscosity under steady shear (Fig. 2a, inset), when the angular frequency (s^−1^) matches the shear rate (s^−1^). This is a clear deviation from the Cox-Merz rule (*i.e.*, angular frequency equates with shear rate when both are expressed in s^−1^ units), which typically applies to polymer solutions^15^. This observation denotes the presence of long-range interactions, aggregation and “enthalpic entanglements”^16^, all of which limit the applicability of conventional spinning techniques when used with nanocellulose hydrogels.

The trends observed for CNF hydrogels suggest that the complex viscosity gives an indication of the behaviour of the steady shear viscosity, even in the case of highly concentrated systems. Thus, even though the complex viscosity of the hydrogels could not be measured accurately at 200 s^−1^, the shear rate used in our spinning system, it was useful in identifying the optimal nanofibril spinnability range. It was clearly shown that hydrogels with intermediate nanofibril concentrations were easier to spin (Fig. 2a). Hydrogels with extreme viscosity, either too low (1% solids) or too high (10% solids) did not spin easily (at least in our spinning system with a shear rate of 200 s^−1^). Interestingly, similar observations hold for the storage and loss moduli of the hydrogels (see Supplementary Figure S1).

### Filament Physical-Mechanical Properties

The spinnable hydrogels (solids content between 2% and 7%) were wet-spun into filaments. These filaments are henceforth denoted according to the type of cellulose nanofibrils: CNF or TEMPO-oxidised CNF (TOCNF) and their solids content in the dope. Images of CNF 2% filament are presented in Fig. 2b between crossed polarisers and in Fig. 2c from scanning electron microscopy (SEM). Physical-mechanical properties of the filaments are summarised in Table 2. As shown in this table, the diameter and coarseness of the filaments were affected by the solids content of the hydrogel dope. An increase in the dope solids content, from 2% to 5–7%, maximised the filament density, reaching values equivalent to that of crystalline cellulose, 1.5–1.6 g/cm^3 ^17. Remarkably, this does not translate into a maximum strength since diluted hydrogels produced superior mechanical strength, as indicated in Fig. 2d–e. Low hydrogel viscosity and apparent density as well as high capacity for fibril alignment upon filament formation appear to coexist in systems with high water volume fraction.

The achieved fibril alignment can be qualitatively appreciated by optical and scanning electron micrographs (see Fig. 2b,c, respectively). The filament displayed different colours between crossed polarisers (Fig. 2b), which results from the birefringence caused by an oriented structure. Fibril orientation in the bulk is confirmed by wide-angle X-ray scattering (WAXS), as will be seen below. Conversely, according to SEM (Fig. 2c), the fibrils on the periphery of the filament appear to distribute randomly, though assembled as aligned grooves, probably formed during drying. Despite the fact that alignment of fibrils on the surface mainly occurs during spinning, the observations also suggests that additional alignment may result upon drying.

Differences in the measured apparent thickness were noted depending on the method used for its measurement (Table 2). The thickness gauge typically gives smaller thickness values compared to those from SEM images. This is most likely due to filament compression under the pressure applied by the thickness gauge. The difference increases with increasing filament porosity, from 10 μm (filaments from CNF 5% and 7%) to 15 μm for CNF 2% and even up to 36 μm for TOCNF 2%. This may result from an increased compressibility of the porous filament structure. Nevertheless, for CNF filaments, the mechanical properties follow the same trend as a function of dope solids content, regardless of the measurement method used. Notably, the apparent thickness measured by the two methods for TOCNF filaments diverge significantly. This is explained by the fact that TOCNF filament sorbs more moisture (and becomes more compressible) in the environmental conditions used in thickness gauge measurements (50% RH, 25 °C), as will be discussed below. Consequently, a lower tensile strength and Young’s modulus are calculated by normalising the mechanical properties of TOCNF filaments with the SEM cross-sectional area. This highlights variations in reported data when hydrophilic materials, such as those based on nanocellulose, are measured using different methods and ambient conditions, which are not cited in most typical cases.

### Fibril Alignment

**Figure 3:**
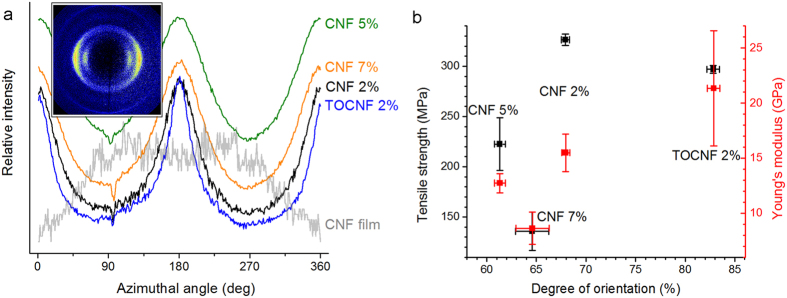
Fibril orientation in wet-spun filaments and its effect on mechanical performance. (**a**) Azimuthal integration of X-ray diffractograms (diffractogram shown for CNF 2% filament in the inset) at a scattering vector of 15.8 nm^−1^. (**b**) Filament tensile strength and Young’s modulus as a function of the degree of orientation. Error bars correspond to the standard deviation among the tested specimens.

In order to further explore the relationship between the properties of the precursor hydrogel and those of the resultant wet-spun filaments, cellulose crystallinity and crystallite orientation were assessed by WAXS. For this purpose, a CNF film cast from a water-acetone mixture was used as a reference for randomly oriented fibrils. The inset of Fig. 3a displays a typical diffractogram obtained for a sample subjected to X-ray irradiation perpendicular to the filament axis (or CNF film plane). The diffractograms were integrated in the radial and azimuthal directions; the cellulose crystallite orientation was quantified based on the azimuthal integration.

According to the obtained X-ray diffractograms (Fig. 3a, inset) the filaments displayed the typical peaks for cellulose I crystals at a scattering vector of 15.8 nm^−1^. (Radial integrations of the diffractograms are plotted in Supplementary Fig. S2) This suggests that CNF crystallinity was not significantly altered by wet-spinning. A distinctive peak is observed for all filaments as a function of the azimuthal angle in the horizontal direction (Fig. 3a), in stark contrast to the intensity distribution of the CNF film. This is taken as further evidence that wet-spinning favours alignment of the fibrils along the vertical (filament axis) direction.

The mechanical strength of the filaments is plotted against the calculated degree of orientation in Fig. 3b. CNF 2% and TOCNF 2% filaments (*i.e.*, the filaments with the highest tensile strength and Young’s modulus) displayed also the highest degree of orientation. However, TOCNF 2% filament appeared significantly more brittle (Fig. 2d), similarly to the CNF 7% filament, which had the most limited performance. These observations indicate that, while mechanical strength correlates with fibril orientation, the filament performance is also influenced by additional hydrogel properties, such as frequency of interfibrillar contacts, which will be discussed below.

### Filament Interactions with Water

**Figure 4:**
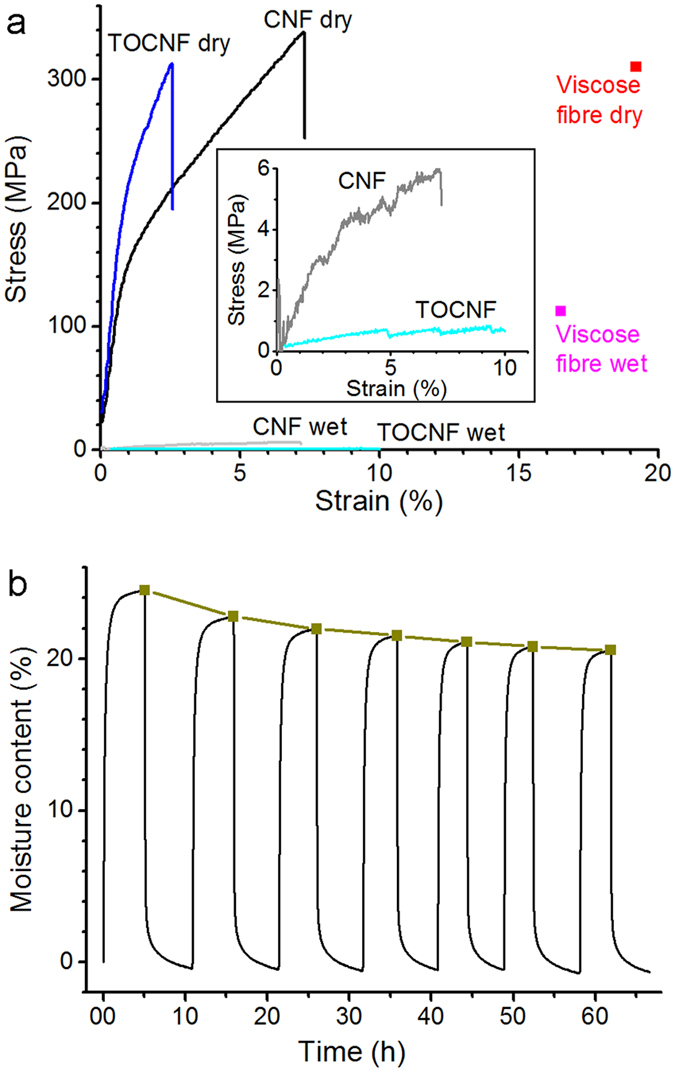
Filament water sorption and its effect on mechanical performance. (**a**) Representative stress-strain curves for CNF 2% and TOCNF 2% filaments in dry condition and after soaking in water for two hours. The drastic reduction in strength illustrates the deleterious effect of water. As a reference, tensile strength and elongation for viscose fibre (coarseness 0.17 g/km, diameter approx. 6 μm) are included^18^. The inset shows the profiles in wet state using reduced plot scales. (**b**) Water sorption analysis for CNF 2% filament. The profile added as a guide to the eye and identified with the square symbols refers to the development of the equilibrium moisture content (EMC) at RH 95%.

The effect of water or moisture on the performance of wet-spun filaments is a critical issue that merits attention. The detrimental effect of water on filaments produced from nanocellulose has not been discussed, except for demonstrations of filament stabilisation towards water through coating^11^ and cross-linking^9^. No experimental data is available on cellulose-water interactions in filaments; even the case of cellulosic fibres is described only to a limited extent. This work assesses the interactions and the effect of water on the properties of CNF and TOCNF filaments. Figure 4a displays representative stress-strain curves for CNF 2% and TOCNF 2% filaments, in both dry and wet conditions. As a reference, the figure includes the tensile strength and elongation of a viscose fibre (coarseness 0.17 g/km, diameter approx. 6 μm)^18^, also prepared by wet-spinning. According to Fig. 4a, CNF filaments can reach a higher tensile strength, but lower maximum strain, than those of the viscose fibre. This highlights the promise of filaments from cellulose fibrils obtained by wet-spinning and coagulation compared to man-made fibres obtained by wet-spinning and regeneration of cellulose derivative solutions. Considering that cellulose orientation can be improved further by changes in the spinning design, it is apparent that wet-spinning of CNF is a competitive alternative. However, as noted, a major drawback that needs to be considered is the lack of strength of CNF filaments in wet conditions. Notably, viscose fibre maintains 36% of its tensile strength when wet^18^, whereas the mechanical performance of CNF filament deteriorates very extensively in the presence of water. This observation is even more dramatic in the case of the TOCNF filaments.

In an attempt to explain the evident decline in mechanical properties under wet conditions, the interactions with water were analysed by means of dynamic vapour sorption (DVS) measurements. A CNF film and wood fibres dried from a water-acetone mixture were used for comparison purposes. An example of a water vapour sorption profile is depicted in Fig. 4b. The equilibrium moisture content (EMC) at 95% RH was determined after several humidity cycles. According to this analysis, the EMC at RH 95% progressively decreased after each cycle, most probably due to irreversible changes in the filament structure, analogous to the so-called “hornification” of cellulosic fibres^19^,^20^,^21^. The extent of hornification was quantified by approximating the EMC progression with the humidity cycles (Fig. 4b in brown) with an exponential function^19^. Here, we use the “% limiting hornification” as the reduction in EMC value obtained by extrapolation of the exponential profile for an infinite number of humidity cycles. The lowest limiting hornification (9%) was obtained for the TOCNF filament. This corresponds to only slightly more than half of the values recorded for all the other samples (between 16 and 17%). This denotes that the humidity cycles hornify TOCNF to a lesser extent compared to the other materials. As such, it reflects the interfibrillar electrostatic repulsion that limits the hornification mechanism or associated effects, including irreversible hydrogen bond formation, pore closure and fibril aggregation^21^.

**Figure 5:**
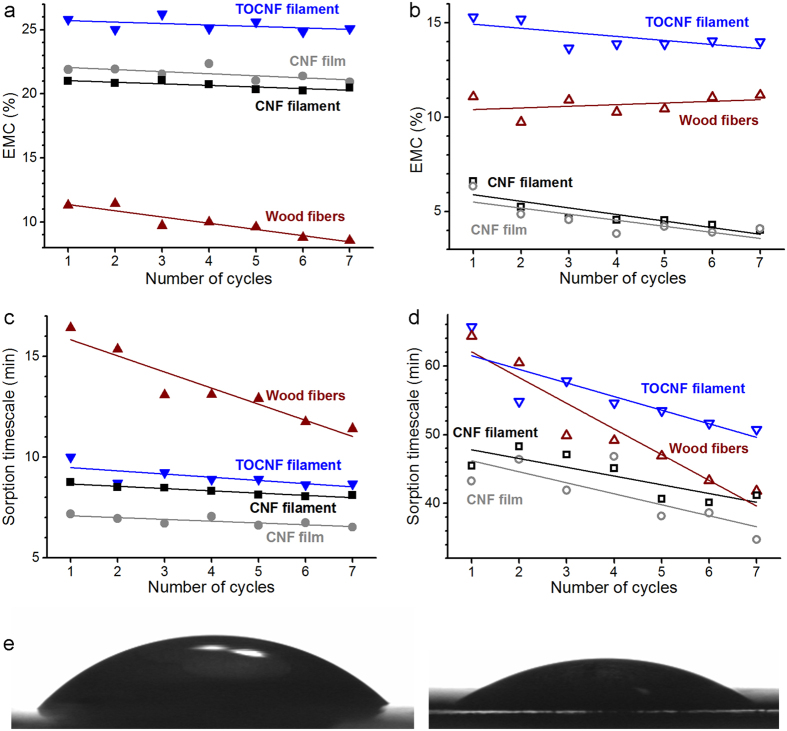
Contribution of two parallel sorption regimes (**a**-**b**) to attain an equilibrium moisture content (EMC) and (**c**-**d**) timescale involved in the process. (**a**,**c**) Correspond to EMC and timescale for the fast sorption regime. (**b**,**d**) Correspond to EMC and timescale for the slow sorption regime. Lines are drawn to guide the eye. (**e**) Photograph of a drop of water on flat films prepared from CNF (left) and TOCNF (right). The TOCNF film contains more moisture than CNF film, as indicated by (**a**-**b**).

Also, EMC and timescales associated with different sorption regimes were determined based on the measured adsorption-desorption profiles according to the parallel exponential kinetics model^22^. These parameters are presented in Fig. 5a–d for each sample, split into fast and slow sorption regimes. Fast sorption refers to sorption occurring at the more exposed sites while slow sorption corresponds to the less accessible sites. For both sorption regimes, EMC and timescale appear fairly similar for the CNF filaments and the film (Fig. 5a–d), regardless of the humidity cycle. This confirms that the moisture sorption behaviour of solvent-exchanged and dried CNF is somewhat unaffected by the preparation method used (wet-spinning or film casting). However, a more significant difference is observed for TOCNF. For any given humidity cycle, the TOCNF filament displayed a higher EMC than any of the other samples (Fig. 5a–b), that is, TOCNF sorbs the highest amount of water. This agrees with the stronger water affinity of TOCNF compared to CNF, as confirmed by water contact angle measurements on corresponding flat films (Fig. 5e): the initial water contact angle of TOCNF (21° ± 5°) is half of that for a CNF film (42° ± 5°). The affinity between TOCNF and water can explain the strong moisture sorption (Fig. 5a-b) and thus the steep decline in the mechanical performance of the filament in wet conditions (Fig. 4a). In addition, it is conceivable that TOCNF is more prone to high moisture adsorption due to its associated high fibril aspect ratio, which results in a relatively larger surface area. Nevertheless, as noted earlier, the high aspect ratio of TOCNF also promotes effective fibril orientation and thus mechanical strength and stiffness (Fig. 3b). This signifies a trade-off between mechanical performance and water stability.

For all the nanofibril samples, the EMC and timescale of the slow sorption processes decrease with the humidity cycles; *i.e.*, mainly the less accessible sorption sites collapse and the remaining ones are occupied faster (Fig. 5b,d). In contrast, the EMC and timescale for fast processes remain fairly independent of the cycle; *i.e.*, more exposed sorption sites maintain their accessibility (Fig. 5a,c). Interestingly, for macroscopic wood fibres, this trend is reversed and most of the hornification is caused by the more accessible surfaces collapsing. In this case, though, also the timescale of the slow sorption processes decreases. This signifies that the closure of the fast sorption sites during hornification makes slow sorption sites more accessible, even though their number density remains constant. The results obtained for wood fibres are consistent with an earlier study reporting the dominant decay of the fast sorption processes and the decrease of both timescales with hornification^19^.

## Discussion

**Figure 6:**
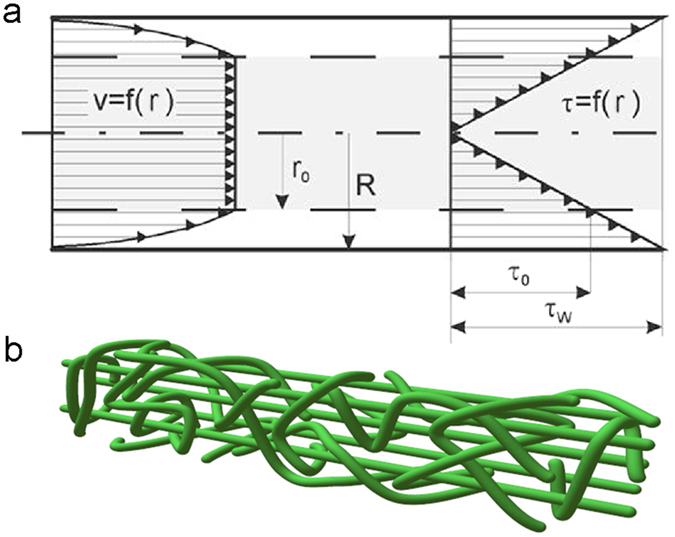
Hypothetical effect of flow regions inside the wet-spinning nozzle. (**a**) Expected flow profile for a shear-thinning fluid with a yield stress in a capillary of radius R (adapted with permission from ref. 37), where v (profile on the left) and τ (profile on the right) are velocity and shear stress in fluid layers at a radial distance r from the centre. r_0_ is the outer radius of the plug flow region; *i.e.*, the radius where τ exceeds the yield stress τ_0_. τ_w_ is the shear stress at the wall, which is proportional to the pressure drop over the capillary and inversely proportional to the capillary length divided by its diameter. (**b**) Graphical representation of the suggested fibril arrangement in the filament. The periphery has aligned randomly due to high shear, while inside, filament comprises partly aligned fibrils owing to traces of orientation created by extensional flow.

The wet-spun filaments were revealed to contain both aligned and non-aligned fibrils (Figs 2b–c and 3). The spatial distribution of aligned and non-aligned fibrils can be further hypothesised to originate from the flow regime and dehydration of the precursor hydrogel before, during and after passing through the wet-spinning nozzle. In the zone of the container approaching the nozzle, the large velocity gradient (*i.e.*, extensional flow) favours fibril alignment. Inside the nozzle, the fibrils are then subjected to laminar shear or plug flow (see Supplementary Discussion), depending on their location (Fig. 6a)^23^,^24^. Negligible spatial variations in velocity occur in the plug flow region (r < r_0_ in Fig. 6a), whereby the effect of shear stress on the fibrils is limited. In these conditions, the initial extensional alignment is gradually offset by Brownian motion^7^, while negligible additional alignment occurs. Outside the plug flow region (r_0_ < r < R in Fig. 6a), the shear stress is larger than the yield stress τ_0_. Yielding under shear has been observed to break the cellulose microfibril network into flocs^25^,^26^. Hence, within this flow region, shear as well as fibril rotation and flocculation have conflicting effects towards fibril alignment. This prevents prominent alignment of individual fibrils on the surface of the filament. In the nozzle exit zone, fibrils may pack into a more oriented structure by the effect of the anti-solvent (acetone) while the filament coagulates and dehydrates, as observed for colloidal rods upon solvent depletion^27^. Similar effect may proceed upon acetone evaporation under drying. Figure 6b includes a speculative explanation of fibril arrangement in the ensuing filament.

The fibril alignment caused by the extensional and shear forces as the fibrils enter the nozzle region will be hereafter referred to as flow-induced alignment. Correspondingly, the packing of fibrils towards an oriented structure upon solvent depletion will be denoted as contact-induced alignment. Both of these orientation mechanisms are necessary for successful wet-spinning of CNF hydrogels. The flow-induced alignment dominates in hydrogels where few interfibrillar contacts occur, whereas contact-induced alignment is favoured at a high osmotic pressure (high fibril electrostatic charge and frequent interfibrillar contacts). A very low or very high density of interfibrillar contacts can disturb contact-induced or flow-induced alignment, respectively, and thus prevent spinning into long filaments.

The frequency of interfibrillar contacts can be quantified using concepts that have been applied to macroscopic fibres, by calculating the crowding number *N* given by equation (1)^28^,^29^.


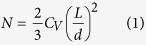


which specifies that *N* grows linearly with the fibre volumetric concentration fraction *C*_*V*_ and the square of *L/d* (the fibre aspect ratio). Equation (1) shows that the calculated crowding number increases linearly with the solids content in the CNF hydrogels that contain fibrils of the same aspect ratio. This effect is more pronounced in the case of TOCNF fibrils since they have a distinctly higher aspect ratio compared to that of CNF (see Supplementary Fig. S3). Accordingly, the square of the aspect ratio of TOCNF was estimated to be over seven-fold higher than that of CNF. Consequently, the crowding numbers of the studied hydrogels can be ranked as follows:


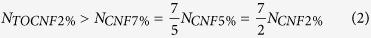


In the case of rod-like colloids, a high volume fraction and aspect ratio induce a high apparent viscosity, frequent contacts between adjacent rods, and a tendency to flocculate^30^. Ostensibly, similar effects apply to cellulose nanofibrils in an aqueous colloidal dispersion. A high crowding number can therefore explain effective orientation combined with brittleness in filaments such as those obtained from TOCNF 2%.

**Figure 7:**
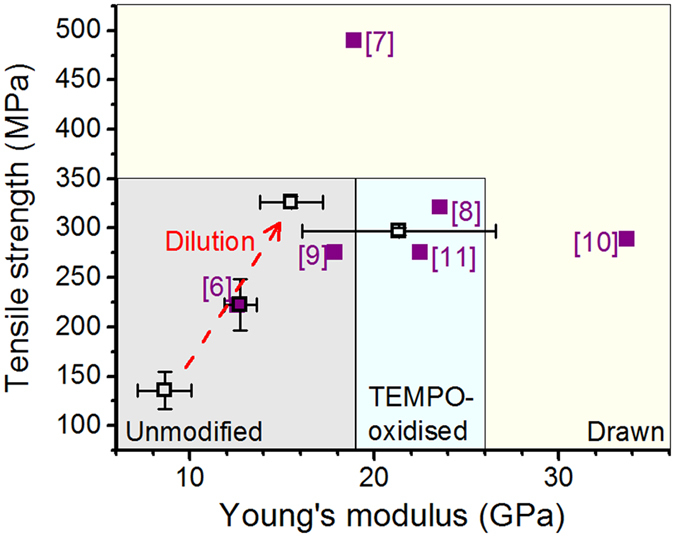
Tensile strength – Young’s modulus plot for cellulose I filaments reported in previous (filled squares) and present (open squares) work. The performance domains achievable with various types of CNF and with drawing are highlighted with different background colour. The effect of CNF hydrogel dilution is illustrated with a dashed arrow. Note the overlap of data points from ref. 6 and CNF 5% filament. Error bars correspond to the standard deviation among the tested specimens.

In the case of TOCNF 2% hydrogel, the high crowding number is combined with relatively low viscosity, which favours both flow-induced and contact-induced alignment. Furthermore, the forces generated by the interfibrillar contacts are strengthened by the elevated osmotic pressure that arises from the carboxylic groups present in TOCNF. In the case of CNF-based filaments, necessary crowding effects to facilitate alignment can only be achieved at a high solids content. Nevertheless, at low hydrogel concentrations, flow-induced alignment improves the quality of the filament, with an optimum at 2% solids content (for the conditions used in our system). Essentially, hydrogel dilution can be used to move filaments diagonally upwards along a tensile strength – Young’s modulus plot (Fig. 7). The positive effect of dilution has also been reported for dry-spun CNF filaments^6^. Furthermore, enhanced fibril surface charge can improve the filament Young’s modulus. Figure 7 also implies that the filaments produced in this work reach or exceed the levels of mechanical performance reported in most of the previous studies. The only reports of significantly higher tensile strength^7^ or Young’s modulus^10^ rely on fibril orientation enhancement via additional extensional forces, which we did not attempt.

A limited drawability of as-spun, wet CNF filaments can be expected due to the poor wet strength of CNF. Conventionally, polymers are drawn during spinning beyond their yield point, up to a strain range where necking occurs^31^. For CNF, only a slight necking phenomenon has been reported^32^. Consequently, full exploitation of these effects remains a challenge in wet-spinning of CNF colloidal dispersions. As a facilitator for drawing, a lubricant or plasticiser could be applied to influence the flow resistance of CNF. Recently, lubrication by grafted PEG was demonstrated to remarkably enhance the orientation in CNF hydrogels stretched into ribbons^4^. Similarly, incorporating lubrication during wet-spinning of CNF could possibly allow for a larger proportion of the fibrils to align inside the filament. However, related effects of such components on water sensitivity need to be considered.

Despite the limited fibril orientation achieved in our experiments, the filaments obtained in this work by wet-spinning displayed a mechanical strength competitive with that of a viscose fibre in dry conditions. Furthermore, our results indicate that filament stability in the presence of water can be achieved by limiting the number of water sorption sites, for example, by decreasing the fibril surface area and electrostatic charge. However, these changes impair fibril alignment and thus the mechanical performance of the filament. For example, the high aspect ratio of TOCNF promotes both high moisture sorption (Fig. 5a-b) and effective fibril orientation and thus mechanical strength and stiffness (Fig. 3b). The trade-off between hydrophilicity and strength of filaments produced from nanocellulose needs to be addressed.

Through DVS studies (see Fig. 5a–d), cellulose nanofibrils were discovered to undergo a different hornification compared to (macroscopic) fibres, likely owing to their structural differences. Contrary to CNF, wood fibres used as a reference in our experiments, are high-order, hierarchical constructions of cellulosic fibrils. In a wood fibre, elementary fibrils assemble first into fibril bundles, which in turn associate to form the plant cell wall according to a controlled binding pattern. This advanced hierarchy provides water sorption sites prone to hornification on many levels – from the more accessible sites to the less accessible ones in the inner regions of the cell wall. Among these, the more exposed sites contribute most to hornification (Fig. 5a in brown). In the case of CNF materials, though, elementary fibrils remain aggregated into bundles but lack a hierarchical assembly. Consequently, the only readily accessible sorption sites are located on external surfaces of the fibril bundles, which undergo negligible hornification (Fig. 5a). The remaining sorption sites susceptible to hornification are located inside the fibril bundles and thus less accessible (Fig. 5b). Moreover, during coagulation, most of the residual water enclosed between the fibrils is exchanged with acetone. Thus, during evaporation, relatively weak capillary forces exist since acetone has a lower surface tension than water. In conclusion, when CNF is forced to aggregate during wet-spinning, the fibrils form a structure that is very different from that of typical fibrils assembled in macroscopic wood fibres.

The structural differences indicated and affecting solvent-exchanged CNF material and wood fibre is akin to the structural change observed upon conventional cellulose polymer processing. When processed via dissolution, wet-spinning and regeneration, cellulose does not reproduce the original crystalline structure during recrystallisation. The major shortcomings of both the dissolution-regeneration and the fibrillation-coagulation routes arise from related limitations upon filament formation: dissolution-regeneration of cellulose leads to filaments with limited mechanical strength, while fibrillation-coagulation of cellulose nanofibrils produces filaments with high water sensitivity. This remains a challenge that needs to be overcome if CNF-based filaments are to be deployed in typical conditions of use.

## Conclusions

We compared unmodified CNF for filament synthesis against TEMPO-oxidised CNF (TOCNF), which was used as a reference. Consideration of hydrogel rheology can be used as a facile way to indicate the spinnability of the nanocellulose dopes. This simple approach is very useful in attempts to scale up the process, to facilitate the determination of the spinning conditions window and to reduce the demand for a large number of more laborious spinning experiments. The results of wet-spinning are ascribed to fibrillary crowding effects and fibril alignment. It was found that the orientation of CNF in wet-spun filaments is induced both by the flow field in the nozzle and by fibril contacts upon solvent removal. A high degree of orientation is favoured under conditions of low hydrogel viscosity and high osmotic pressure. The CNF-based filaments obtained in this work by wet-spinning displayed a higher mechanical strength than that of a viscose fibre in dry state. Our results indicate that filament stability in the presence of water can be achieved by limiting the number of water sorption sites, for example, by decreasing the fibril surface area and electrostatic charge. However, these changes impair fibril alignment and thus the mechanical performance of the filament. The trade-off between hydrophilicity and strength of filaments produced from nanocellulose needs to be addressed to warrant their deployment and to achieving the desired performance of super-filaments (filaments with highest possible strength).

## Methods

### Cellulose Nanofibrils

Never-dried, bleached birch fibres were refined with a Voith LR 40 laboratory refiner for 12 minutes at a solids content of 3% in deionised water at the following refining conditions: refiner speed 200 rpm, specific edge load 0.5 J/m, net specific energy 294 kWh/t and net refining power 1.39 kW. In order to obtain a reference sample with increased surface charge and aspect ratio, non-refined never-dried bleached birch fibres were oxidised with 2,2,6,6-tetramethylpiperidine 1-oxyl (TEMPO). Alkaline TEMPO-NaBr-NaClO system was used at pH 10 for the TEMPO-oxidation. In short, the fibres were dispersed in deionised water into a solids content of 1%, followed by addition of the oxidation chemicals: TEMPO (0.13 mmol) and NaBr (4.65 mmol per gram of fibres). The pH of the solution was adjusted close to 10 in order to impregnate the oxidation chemicals in the fibre structure through fibre swelling prior to addition of NaClO solution (active chlorine content of 5 mmol per gram of pulp) in five equally sized aliquots. After oxidation, the fibres were washed with deionised water, incubated at pH 2 for half an hour and washed again with deionised water until low conductivity. Thus, carboxylated fibres were obtained in the acidic form. A carboxylic group content of 1.2 mmol/g based on dry mass was attained for the batch used for rheological characterisation, atomic force microscopy, and contact angle measurements. The batch used for spinning and filament characterisation contained 1.36 mmol/g of carboxylic groups. Before the fluidisation, the TEMPO-oxidised fibres were diluted with deionised water and titrated with NaOH to a pH of 8.5 for changing the carboxyls of TEMPO-oxidised fibres to sodium form.

The refined and TEMPO-oxidised wood fibres were fluidised by a high-pressure microfluidiser (Microfluidics Corp., USA). The bare and carboxylated fibres, diluted in deionised water (2% solids content) were passed through a chamber pair of 200 and 100 μm under a pressure of 2000 bar for six and one times, respectively. The ensuing fibril diameters of prepared CNF gels were qualitatively evaluated by atomic force microscopy (AFM, see Supplementary Fig. S3). Prior to experiments, the obtained pristine CNF hydrogel was diluted with deionised water or (ultra)centrifuged to prepare a series of precursor hydrogels at different solids contents from 1% to 10%. Based on the mechanical performance of the filaments spun from these samples, a hydrogel solids content of 2% was selected for further studies on the effect of cellulose surface charge. The TOCNF hydrogel was therefore used for comparison without further adjustment of the solids content.

### Wet-Spinning

All hydrogels were spun through a straight steel nozzle (diameter 1.3 mm, length 10.5 cm) to an acetone bath at a speed of 7.5 m/min (10 ml/min). The spun filaments were allowed to coagulate in the bath for approximately five minutes. After coagulation, the filaments were dried in air while the filament ends were immobilised. Otherwise, under free-drying (without ends fixed), filaments were observed to curl. The contraction upon drying has been thoroughly studied in the case of cellulosic paper^33^. In the case of single cellulose aggregate fibrils, no apparent longitudinal shrinkage occurs upon drying under decreasing humidity^34^. Consequently, little longitudinal contraction was expected from perfectly aligned CNF. However, the alignment of CNF achieved in the nozzle relaxes rapidly due to Brownian motion^7^. Because of the heavy radial shrinkage of non-oriented fibrils, filaments contracted along all directions during drying unless they were longitudinally restrained. A video of the filament preparation can be found in the Supplementary Information.

### Film Casting

In order to compare the properties of the spun filaments to those of their precursor materials upon exchange with the same solvent, CNF 2% and TOCNF 2% hydrogels were diluted with an excess of acetone. The obtained dispersions were cast on a dish and let dry in air overnight in a fume hood for a minimum of three hours. In the case of TOCNF, drying only occurred over several days in fume hood. Similar solvent exchange and drying were performed also on the refined wood fibres that were used without fluidisation.

### Rheological Characterisation

Rheology of the hydrogels was analysed with an MCR 300 rheometer (Anton Paar, Austria). Plate-plate geometry was used with a steel plate (diameter 25 mm) and a gap of 1 mm. For apparent viscosity measurements under steady rotation, serrated plates were used to avoid wall slip. However, only the most diluted hydrogels (1–2% solids content) were suitable for the apparent viscosity measurement. At higher solids contents, the rotation caused the sample to fracture and/or escape the gap. The viscosity was measured as a function of shear rate, varying the shear rate between 0.1 and 1000 s^−1^.

For oscillatory measurements, the linear viscoelastic region of the hydrogels was first determined by measuring the storage and loss moduli as a function of strain amplitude in the amplitude range of 0.01–100%. For all the hydrogels, these moduli remained independent of strain amplitude around an amplitude of 0.1%. Accordingly, 0.1% was selected as the amplitude for frequency sweeps. Angular frequency of the oscillation was varied between 0.1–5 s^−1^, and its effect was measured on the storage and loss moduli as well as on the complex viscosity.

### CNF imaging and filament morphology

CNF and TOCNF hydrogels were diluted to final concentrations of 5 mg/l and 8 mg/l, respectively. 25 μl of each dispersion was cast on a mica support and dried for an hour at 50 °C. The dry samples were imaged with AFM (Nanoscope IIIa Multimode scanning probe microscope from Digital Instruments Inc., USA). Images were obtained by using tapping mode in air with silicon cantilevers. For filament imaging, SEM was used with magnifications 10300x and 35490x (Zeiss Sigma VP scanning electron microscope, Carl Zeiss Microscopy Ltd, Cambridge, UK). The operating voltage was either 3 kV or 2 kV and the working distance either 2.5 mm or 2.6 mm. Prior to imaging, the sample was sputtered with carbon followed by gold-palladium coating. Optical microscope image between crossed polarisers was obtained with a polarising microscope Leica DM4500 P equipped with a Leica DFC420 camera (Leica Microsystems, Germany).

### Physical properties

Apparent filament density was estimated by weighing an approximately 1 cm long filament, measuring its length and diameter based on the respective SEM image and assuming a circular cross-section. This apparent density measurement was repeated at least five times for each sample. The density values were converted to coarseness (mass per unit length) by assuming a constant average cross-section along the length of the filament, as measured by SEM. The apparent porosity was calculated by comparing the apparent density of the filament to the density of pure cellulose, according to equation (3).


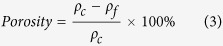


where *ρ*_*c*_ represents the density of crystalline cellulose (1.5–1.6 g/cm^3^)^17^ and *ρ*_*f*_ the apparent density of the filament.

### Mechanical Strength

Filament mechanical properties were characterised with an MTS 400 tensile tester (MTS Systems Corp., USA) by applying a load cell of 50 N, a gauge length of 30 mm and a cross-head speed of 30 mm/min. A relative humidity (RH) of 50% and a temperature of 25 °C were maintained during the measurements. The cross-sectional areas of the filaments were estimated by assuming a circular cross-section and measuring filament diameters by a thickness gauge under a low and constant pressure, according to the international standard regarding thickness of paper and board (ISO 534). RH conditions during the thickness measurement were maintained the same as those during mechanical testing (RH 50% and 25 °C). Before mechanical testing, filaments were equilibrated for two hours at RH 50%. We noted from DVS studies (see Fig. 4b) that the filaments undergo most of their moisture exchange during this time period. Henceforth, we refer to the conditions specified above as “dry” conditions. Before the tests in wet state, filaments were immersed in deionised water for two hours. The wet filament diameter was estimated by comparing the diameters of ten specimens of dry and wet filaments under optical microscope. The average ratio between the wet and dry diameters (1.4 for CNF or 3.6 for TOCNF) was used to multiply the dry diameter measured by the thickness gauge under the tensile testing conditions. Ten repetitions of the tensile test were performed for each sample.

### Fibril alignment

Fibril orientation in the filaments was examined via Wide Angle X-ray Scattering (WAXS) by using a MicroMax-007 X-ray generator (Rigaku, Japan) operating at a wavelength of 1.54 Å, with a beam size of 120 μm, exposure time of 10 minutes and sample-to-detector distance of 200 mm. Diffraction patterns were collected with a Mar345 imaging plate detector. Background noise was subtracted from all the samples. Measurements were repeated at three different locations for each sample. The obtained intensity distribution profile was summed in radial direction to follow differences in crystallinity between samples. Following the highest peak in this distribution (scattering vector 15.8 nm^−1^), azimuthal intensity distribution profile was used to calculate the degree of orientation (*f*_*c*_) according to equation (4).


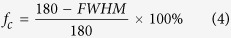


where *FWHM* is the full width at the half maximum (in degrees) of a peak in the azimuthal intensity distribution profile.

### Water Contact Angle

The initial, static water contact angles of CNF and TOCNF films cast from water-acetone mixture were measured with a CAM 200 optical contact angle meter (KSV Instruments, Finland) at room temperature. Drops of approximately 7 μl were deposited on the films and imaged immediately. Contact angles were computed based on the images. Measurements were performed three times for both samples.

### Interactions with moisture

Water vapour sorption isotherms of the filaments were determined by using the dynamic vapour sorption method (DVS Intrinsic apparatus, Surface Measurement Systems, UK). In addition, a CNF film and wood fibres dried from acetone were tested as references. The filaments were cut in 5 mm pieces to fit the sample pan. The pan was loaded with approximately 4 mg of the sample and hung from a microbalance in a climate-controlled chamber. The RH inside the chamber was first decreased to 0% until the sample mass became stable (change in mass below 0.002%/min over a period of 10 minutes). After this, the relative humidity (RH) was increased to 95% until the sample mass was stabilised similarly. RH was cycled in this way between 0% (moisture desorption) and 95% (moisture adsorption) for seven times in total. The relative increase in sample mass compared to the mass after the first drying at 0% RH corresponds to the moisture content adsorbed by the sample at 95% RH.

The results were analysed by fitting an exponential function to the EMC achieved at the end of each cycle at 95% RH, reflecting the effect of the so-called “hornification” on the sorption capacity of the sample^19^. Based on this fitting, the limiting hornification (*LH*) can be estimated according to equation (5).


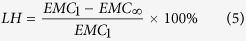


where *EMC*_*1*_ corresponds to the EMC at the end of the first cycle at RH 95% and *EMC*_*∞*_ at the end of the n^th^ cycle when n approaches infinity. *EMC*_*∞*_ is approximated according to the exponential fit of the EMC measured at the end of each of the seven cycles. It should be noted that the EMC values obtained by DVS contain the contribution of external surfaces, which are not susceptible to hornification. Consequently, equation (5) only determines the limiting hornification as a percentage of total initial sorption capacity, even considering those sorption sites that would not partake in hornification effects.

In addition, in order to evaluate the nature of the sorption phenomena, each adsorption cycle was approximated with the parallel exponential kinetics model^22^. This model assumes that the moisture content (*MC*) as a function of time (*t*) at a certain RH can be approximated by equation (6).


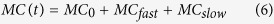


where *MC*_*0*_ is constant and *MC*_*fast*_ and *MC*_*slow*_ represent the contributions of fast and slow sorption processes, respectively, expressed as


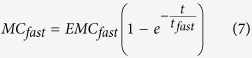



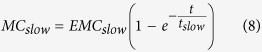


where *EMC*_*fast*_ and *EMC*_*slow*_ correspond to the moisture sorption capacity at fast and slow sorption sites, respectively. Similarly, *t*_*fast*_ and *t*_*slow*_ express the timescales of the fast and slow sorption processes. Here, fast and slow sorption refer to sorption occurring at more and less exposed sites, respectively.

## Additional Information

**How to cite this article**: Lundahl, M. J. *et al*. Strength and Water Interactions of Cellulose I Filaments Wet-Spun from Cellulose Nanofibril Hydrogels. *Sci. Rep.* **6**, 30695; doi: 10.1038/srep30695 (2016).

## Supplementary Material

Supplementary Information

Supplementary Information

## Figures and Tables

**Figure 1:**
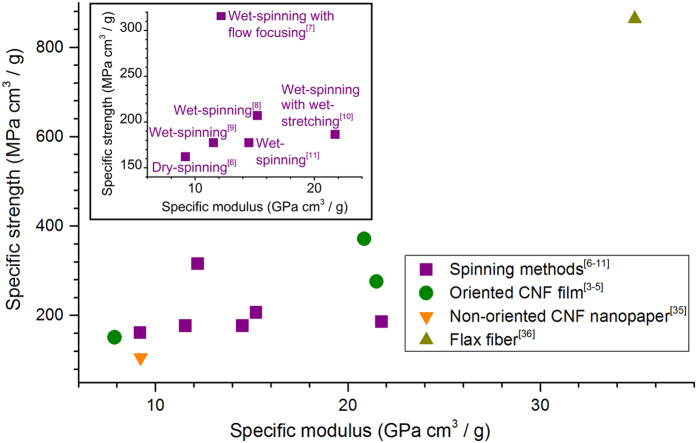
Ashby plot of the specific strength *versus* specific elastic modulus for CNF-based materials obtained by various alignment methods. Reference materials are included with different degrees of alignment, from nanopaper with randomly oriented CNF^35^ to highly oriented flax fibres^36^. The inset shows only the cellulose I filaments prepared by spinning. Note: If not provided in the original source, a cellulose density of 1.55 g/cm^3^ (corresponding to that of crystalline cellulose)^17^ was assumed in order to estimate the properties from the reported data.

**Table 1:** Key differences between conventional polymer and CNF spinning processes.

	Polymer spinning	CNF spinning
Dope	Solution, dispersion or melt of individual polymer chains (high aspect ratio)	Dispersion of colloidal fibrils or semi-crystalline polymer bundles (low aspect ratio)
Alignment mechanism	Flow and drawing; high drawability	Flow (with flow focusing^7^ or wet-stretching^10^); low drawability
Filament stabilisation mechanism	Polymer crystallisation	Interfibrillar interactions

**Table 2:** Average values of physical and mechanical properties of filaments produced by wet-spinning of cellulose nanofibril hydrogels (CNF and TOCNF).

Property	Filament			TOCNF 2%
	CNF 2%	CNF 5%	CNF 7%	
Apparent thickness (thickness gauge), μm	106 ± 5	171 ± 20	225 ± 35	80 ± 4
Apparent thickness (SEM), μm	121 ± 4	181 ± 9	235 ± 11	116 ± 14
Apparent density, g/cm^3^	1.20 ± 0.29	1.58 ± 0.07	1.51 ± 0.06	1.06 ± 0.24
Apparent coarseness, g/km	1.81 ± 0.90	4.01 ± 0.60	6.56 ± 0.88	1.19 ± 0.52
Apparent porosity, %	23 ± 18	∼0	∼0	32 ± 15
Tensile strength, MPa	326 ± 6	223 ± 26	136 ± 19	297 ± 4
Specific strength, MPa cm^3^/g	290 ± 75	142 ± 23	91 ± 17	296 ± 71
Young’s modulus, GPa	15.5 ± 1.7	12.7 ± 0.9	8.6 ± 1.5	21.3 ± 5.5
Specific modulus, GPa cm^3^/g	14.1 ± 4.8	8.1 ± 0.9	5.8 ± 1.3	22.4 ± 10.0
Strain at break, %	6.9 ± 0.9	8.4 ± 2.1	3.7 ± 1.4	2.8 ± 0.5

Error values are estimated based on the standard deviation among the tested specimens.
