# Supplementary material for: Strength and Water Interactions of Cellulose I Filaments Wet-Spun from Cellulose Nanofibril Hydrogels
Source: Sci Rep. 2016 Jul 28;6:30695. doi: 10.1038/srep30695 (PMC4964603; doi:10.1038/srep30695)
Supplement: Supplementary Information [file srep30695-s1.pdf]

Supplementary Information

Strength and Water Interactions of Cellulose I Filaments Wet-Spun from Cellulose Nanofibril Hydrogels

Meri J. Lundahl[*,†], A. Gisela Cunha[†], Ester Rojo[†], Anastassios C. Papageorgiou[#], Lauri Rautkari[†], Julio C. Arboleda[†], Orlando J. Rojas[*,†]*

[†]Aalto University, Department of Forest Products Technology. P.O. Box 16300, 00076 Aalto, Finland

[#]Turku Centre for Biotechnology, University of Turku and Åbo Akademi University, 20520 Turku, Finland

Corresponding Authors: (*) E-mail: meri.lundahl@aalto.fi (M.J.L.), Phone: +358 40 526 0787; *E-mail: orlando.rojas@aalto.fi (O.J.R.), Phone: +358 50 5124227.

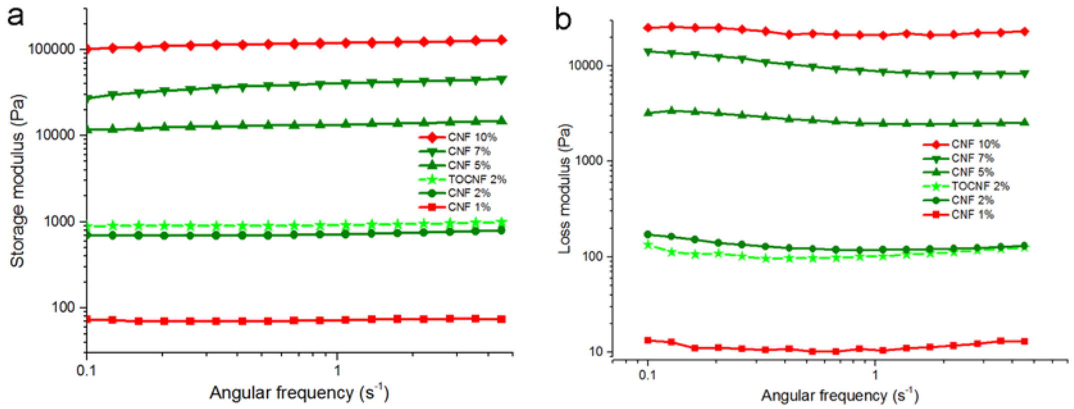

Supplementary Figure S1. (a) Storage and (b) loss moduli as a function of angular frequency for CNF hydrogels at varying solids contents. The spinnability of the hydrogels is indicated using a colour code: green for conditions yielding filaments with continuous length > 5 cm and red for filaments < 5 cm.

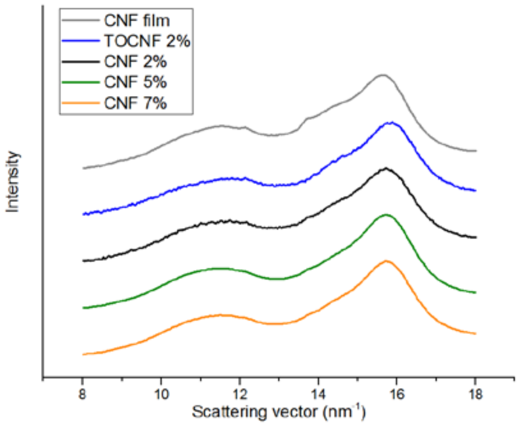

Supplementary Figure S2. Radial integration of the scattered X-ray intensity as a function of the scattering vector.

Supplementary Discussion. Reynolds number of CNF hydrogel in the wet-spinning nozzle.

In order to determine the flow characteristics of CNF in the wet-spinning nozzle, Reynolds number (Re) was calculated according to equation (S1)

$$Re = \frac{\rho \, v \, d}{\mu} \quad \text{(S1)}$$

where ρ = hydrogel density (assumed as similar to the density of water), v = flow velocity, d = nozzle diameter and μ = hydrogel viscosity at the applied shear rate. The applied shear rate ($\dot{\gamma}$) was estimated by equation (S2)

$$\dot{\gamma} = \frac{2 \, v}{d} \quad \text{(S2)}$$

CNF 2% hydrogel was used for this calculation because of its lowest apparent viscosity (1.37 Pa s) at the system shear rate (approximately 200 s^{-1}) among the spinnable samples. Thus, it has the highest Reynolds number of 0.12. Even this value is clearly below the maximum Reynolds number for laminar flow in a cylindrical pipe (2300). The flow of all the tested hydrogels is, therefore, laminar in the wet-spinning nozzle.

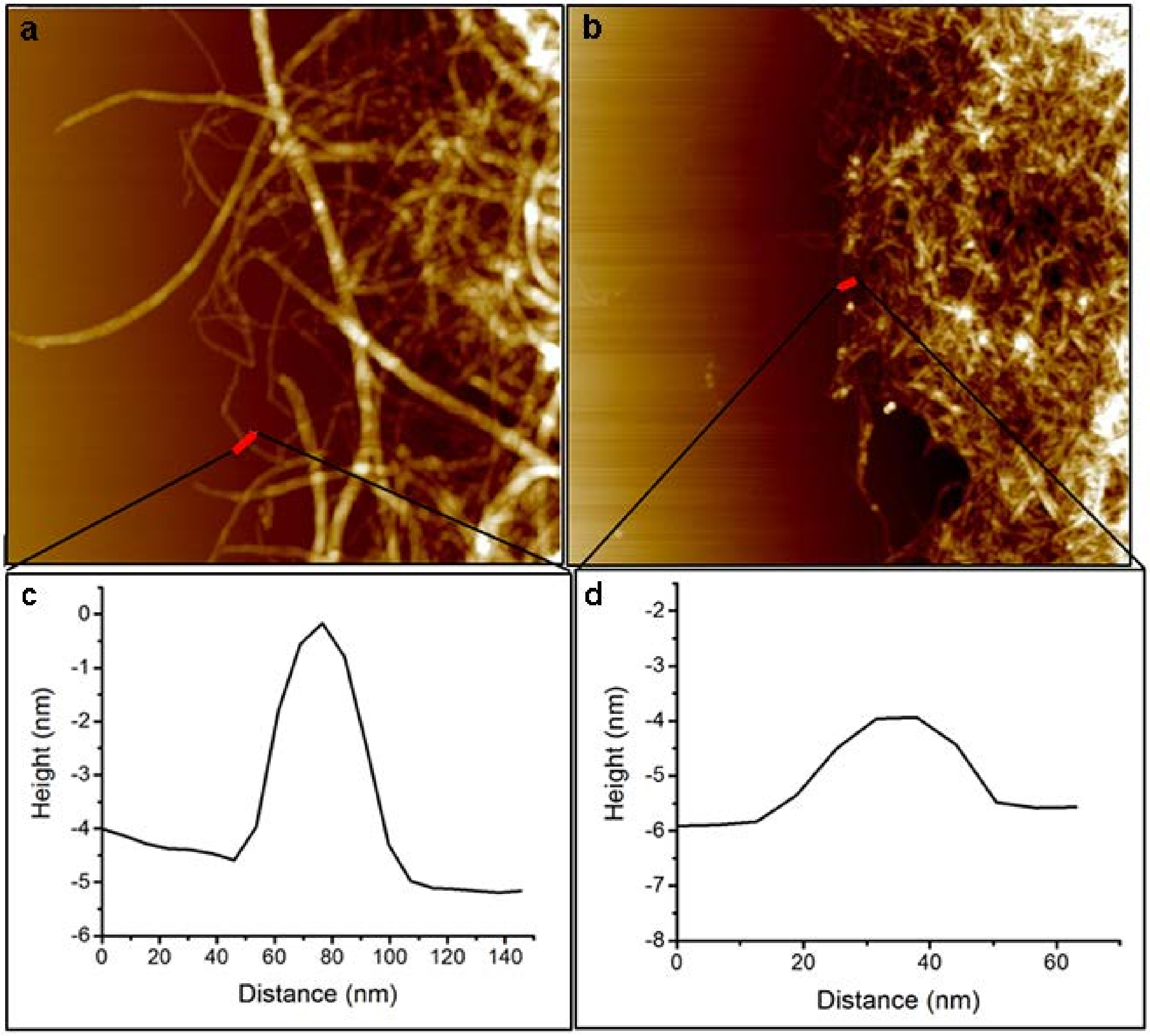

Supplementary Figure S3. AFM height images (3 µm x 3 µm) of (a) CNF and (b) TOCNF hydrogels showing individual fibrils. The single fibril height profiles in (c) for CNF and (d) for TOCNF illustrate the differences in the fibril diameters. Based on these profiles, unmodified fibrils have more than double the diameter of TEMPO-oxidised fibrils.

A **supplementary video** illustrating the sample preparation process can also be found at http://www.nature.com/srep. The video is published with permissions from Eeva Suorlahti and Tekes.
